# Leveraging Google Earth Engine for a More Effective Grassland Management: A Decision Support Application Perspective

**DOI:** 10.3390/s24030834

**Published:** 2024-01-27

**Authors:** Cecilia Parracciani, Daniela Gigante, Federica Bonini, Anna Grassi, Luciano Morbidini, Mariano Pauselli, Bernardo Valenti, Emanuele Lilli, Francesco Antonielli, Marco Vizzari

**Affiliations:** Department of Agricultural, Food, and Environmental Sciences, University of Perugia, 06121 Perugia, Italydaniela.gigante@unipg.it (D.G.); federica.bonini@studenti.unipg.it (F.B.); anna.grassi@studenti.unipg.it (A.G.); luciano.morbidini@unipg.it (L.M.); mariano.pauselli@unipg.it (M.P.); bernardo.valenti@unipg.it (B.V.); emanuele.lilli@studenti.unipg.it (E.L.); francesco.antonielli@studenti.unipg.it (F.A.)

**Keywords:** grassland, grazing management, Web-GIS, Google Earth Engine (GEE), Sentinel-2, harmonic modeling, EVI, decision support systems

## Abstract

Grasslands cover a substantial portion of the earth’s surface and agricultural land and is crucial for human well-being and livestock farming. Ranchers and grassland management authorities face challenges in effectively controlling herders’ grazing behavior and grassland utilization due to underdeveloped infrastructure and poor communication in pastoral areas. Cloud-based grazing management and decision support systems (DSS) are needed to address this issue, promote sustainable grassland use, and preserve their ecosystem services. These systems should enable rapid and large-scale grassland growth and utilization monitoring, providing a basis for decision-making in managing grazing and grassland areas. In this context, this study contributes to the objectives of the EU LIFE IMAGINE project, aiming to develop a Web-GIS app for conserving and monitoring Umbria’s grasslands and promoting more informed decisions for more sustainable livestock management. The app, called “Praterie” and developed in Google Earth Engine, utilizes historical Sentinel-2 satellite data and harmonic modeling of the EVI (Enhanced Vegetation Index) to estimate vegetation growth curves and maturity periods for the forthcoming vegetation cycle. The app is updated in quasi-real time and enables users to visualize estimates for the upcoming vegetation cycle, including the maximum greenness, the days remaining to the subsequent maturity period, the accuracy of the harmonic models, and the grassland greenness status in the previous 10 days. Even though future additional developments can improve the informative value of the Praterie app, this platform can contribute to optimizing livestock management and biodiversity conservation by providing timely and accurate data about grassland status and growth curves.

## 1. Introduction

Grasslands are one of the earth’s most significant land cover types for human well-being and livestock, encompassing approximately 40% of its surface and 69% of its agricultural land area [1,2]. Furthermore, agriculture plays a pivotal role in the European Union, with approximately 180 million hectares, accounting for 42% of the total land area [3,4]. A considerable portion of this land is dedicated to grasslands [4], serving as invaluable pastureland for sustaining livestock farming by providing essential feed resources [3]. In addition, natural and secondary grasslands provide humanity with diverse ecosystem services [1,5,6,7,8,9] while also serving as crucial global biodiversity reservoirs, ranking among the most species-rich plant communities worldwide [1,10,11]. Despite this, it is essential to highlight that a significant portion of these grasslands has been degraded or somewhat altered from their balanced condition. Approximately half of the world’s grassland areas have experienced degradation due to various factors such as overgrazing, inappropriate land management practices, and climate change [1,12]. In Europe, many open agroecosystems, particularly semi-natural grasslands, are at risk due to changing land use and abandonment, which threaten their biodiversity and ecosystem services [3,4,13]. These dynamics have generated severe landscape composition and configuration alterations along the urban–rural gradients [14,15]. Annex 1 of the European Council Habitat Directive of 1992 (Directive 92/43/EEC) listed habitats considered of European importance due to their biodiversity value, including different types of pasture and grassland habitats under threat [3]. Human activity stands as the foremost driver of grassland degradation. Specifically, disturbance from overgrazing reduces vegetation cover, making grasslands more susceptible to soil erosion [1,13].

Conversely, in numerous high-income countries, there has been a noticeable trend toward adopting more intensive cattle production systems for meat and dairy, resulting in a decline in the utilization of extensive, grazing-based livestock systems [16]. In particular, the cessation of extensive breeding in semi-natural grasslands, driven by huge trends of land abandonment in Europe during the 20th century, has resulted in grassland degradation and reduction due to the natural processes of encroachment of shrub and woody vegetation [1,13,17]. Both over- and under-exploitation of grassland-based systems lead to a progressive alteration in the plant community composition, resulting in the loss of grasslands and the establishment of other land cover types [18,19,20].

Gaining insight into the dynamics of grassland ecosystems in space and time is essential for evaluating the efficiency of protective measures and facilitating sustainable farming practices [21]. In this context, some studies have already pointed out how essential it is to enhance the monitoring and management of grazed vegetation and the monitoring of grazing animals (e.g., [22,23]). In this regard, satellite remote sensing presents numerous advantages, offering a powerful and efficient means of cost-effective, timely, and reproducible vegetation analysis [7,22]. One of the significant advantages is its ability to cover extensive areas, providing a macroscopic view of grassland habitats [7]. This comprehensive approach enables the identification of ecological patterns and trends over time, assisting in understanding the dynamics of these ecosystems and the factors influencing their stability. Remote sensing enables the systematic monitoring of large-scale vegetation dynamics, the tracking of changes in plant communities, assessment of biodiversity indicators and indices, and the monitoring of ecosystem health over extended periods [7]. This invaluable tool empowers decision-makers and the scientific community with data to inform land management practices, environmental policies, and conservation efforts. Vegetation indices, in particular, are combinations of digital bands in remote sensing data [24] and have proven to be valuable tools in agroecosystems for indirectly estimating various biophysical vegetation properties [25]. The Enhanced Vegetation Index (EVI) is one of the most used vegetation indices and is particularly significant in grassland research [7,26]. Furthermore, the European Space Agency’s (ESA) Sentinel-2 constellation has significantly enhanced precision techniques for farming practices [27]. This is attributed to the satellites’ frequent revisit time and high spatial and spectral resolution and the open policy adopted for data access.

Web-based geographic information systems (Web-GISs) offer the capabilities to enhance effective communication and collaboration among users by sharing spatial data [28]. In the context of technological progress and geographic information, the opportunities to develop and utilize web-enabled decision support systems (DSSs) for sustainable grassland management have significantly increased [29]. The primary objective of most of these tools is to augment the available information and data related to grassland management [23,29]. By harnessing the power of GIS and integrating various datasets [30], these tools can offer farmers and land managers a comprehensive understanding of their grassland resources [23,29]. For example, the Grazplan Web-GIS assists decision-makers in the Australian agricultural sector, helping them make informed and optimized decisions related to grazing management, pasture utilization, and livestock production [31]. Although Grazplan incorporates a vast array of diverse data that could offer advantages over more straightforward tools, it still relies on three distinct paid software components. The PastureBase Web-GIS offers similar free support to decision-makers in Ireland, empowering them with information regarding grassland management, grazing rotations, and livestock performance [29].

Recent advances in information technology and cloud-based platforms offer potential solutions to the problems of data storage, processing, and accessibility [32]. Google Earth Engine (GEE) has recently been at the forefront of remote sensing big data processing [33,34,35,36]. GEE is a cloud-based platform developed by Google that enables the analysis and visualization of large-scale geospatial datasets and the development of customizable web-based, dynamic, shareable applications accessible from a browser [25,32,37]. These apps allow users to present their geospatial data in an interactive and user-friendly manner, making it easier to communicate complex analyses and findings with a broader audience. GEE provides a wide range of tools, libraries, and application programming interfaces (APIs) to facilitate the creation of sophisticated applications [33,37].

Regarding the sustainable use of grassland, ranchers and grassland management authorities face challenges in effectively controlling herders’ grazing behavior and grassland utilization due to underdeveloped infrastructure and poor communication in pastoral areas [23]. As emphasized by Li et al. [23], a cloud-based grazing management and decision system can address this issue and promote sustainable development. These systems will enable rapid and large-scale monitoring of grazing behavior, grassland growth, and utilization, providing a basis for decision-making in managing grazing and grassland [23,29]. 

In the depicted context, our work aligns with the need for new user-friendly digital tools for livestock management and grassland monitoring and conservation and is part of the broader framework of the LIFE IMAGINE project funded by the European Union (LIFE IMAGINE UMBRIA; LIFE19IPE/IT/000015; www.lifeimagine.eu, accessed on 18 January 2024). This comprehensive initiative incorporates various targeted actions aimed at conserving and preserving the secondary grasslands of Umbria, Italy.

The main objective of this research is to develop a Web-GIS application in GEE capable of providing quasi-real-time geographic information to all project stakeholders (and, hopefully, all the region’s potentially interested parties) to support knowledge-based management decisions about grassland exploitation and conservation. In this context, the Web-GIS should allow for more precise vegetation information over time and space for managing grazing livestock and supporting the sustainable use of grassland habitats. 

The Web-GIS application was named “Praterie”, meaning “grasslands” in Italian. After defining a study area coherently with the general project’s objectives, with the help of various stakeholders, we identified the user requirements of the Praterie app. The subsequent steps were oriented to the definition of the methodological approach, code design, and implementation in GEE.

## 2. Materials and Methods 

### 2.1. Study Area

**Figure 1:**
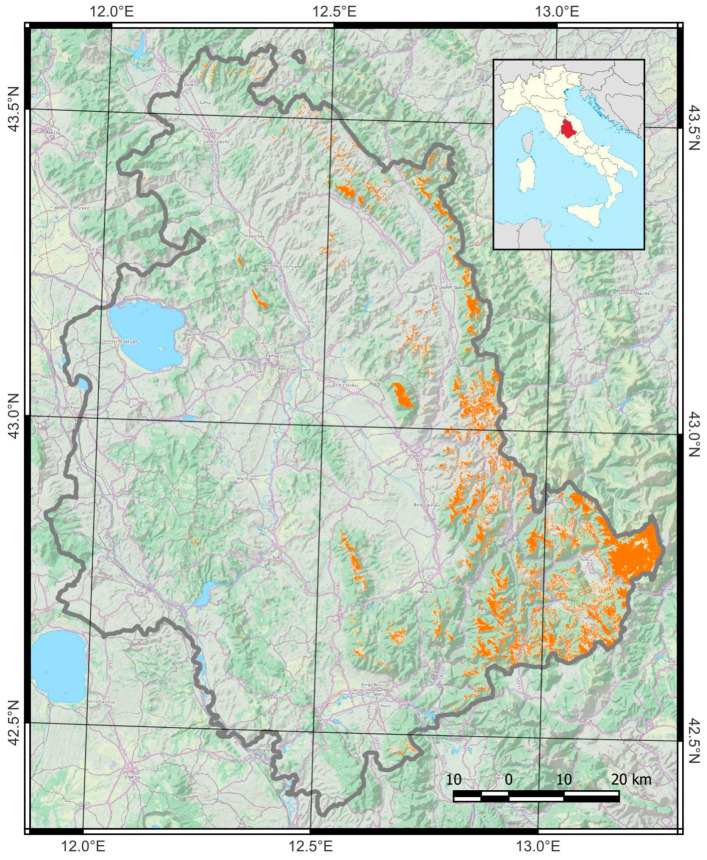
Location of Umbria in Italy (upper-right map, the Umbria region is in red). The orange pixels represent the study area. The regional boundaries of Umbria (dark grey) are also shown. Background: OSM Landscape base map including relief.

The study area is located within the Umbria region of Italy and spans about 385 square kilometers. It was identified by selecting regions classified as grassland under category 30 in the ESA World Cover 2021 [38] and at an elevation of 700 m above sea level or higher, as depicted in Figure 1. This threshold was defined to include Umbria’s most important secondary grassland habitats, most belonging to the Natura 2000 network. Secondary grassland habitats in Umbria mainly correspond to the Annex I types 6210 and 6230*, which may include small patches of 6110* and 6220* (Directive 92/43/EEC). Habitat type 6210 consists of semi-natural dry grasslands and scrubland facies on calcareous substrates, with plant communities generally assigned to the phytosociological class *Festuco valesiacae*-*Brometea erecti* Br.-Bl. & Tüxen ex Br.-Bl. 1949 [39]. Habitat type 6230* consists of closed, dry or mesophile, perennial, acidophilic *Nardus stricta*-dominated grasslands developed on siliceous or decarbonated substrates in mountain areas, including plant communities in the central and southern Italian peninsula assigned to the phytosociological class *Ranunculo pollinensis*-*Nardion strictae* Bonin 1972 [39].

The study area includes many various-sized portions mainly located across the Umbrian Apennines, which experience a Mediterranean climate with a mix of continental influence. This region features hot, dry summers and cold, wet winters. The average temperature in the summer ranges from 20 °C to 30 °C, whereas winter temperatures can drop to −3 °C. The region also receives substantial rainfall (yearly average of 1100 mm), particularly in the winter and spring. The higher altitudes of the mountains are often covered with snow during winter. The population is sparse due to its rugged terrain, lack of urban development, and demographic decline due to socioeconomic dynamics. The area is primarily rural, with small villages and towns scattered throughout the mountains.

### 2.2. Definition of User Requirements

The user requirements of the Praterie app were defined according to the needs expressed by interviews with livestock experts, geo-botanists, and livestock farmers in the study area. The primary needs expressed by this panel were the following: (a) Every user should be able to access the Praterie platform from their browsers without requiring any specific GIS software or technical skills; (b) Praterie must display the locations, timing, and expected vegetative growth and related timing in the grassland areas for the upcoming vegetative cycles of the current year; (c) Praterie must display the vegetation trends over time for different types of areas of interest (in our case, for points or areas chosen by the user); (d) Praterie must display the average observed greenness over the last 10 days, providing an insight into the current state of grassland canopy; (e) to enhance the usability of Praterie, it must display the boundaries of the Natura 2000 protected areas; and (f) Praterie must allow users to choose the visualization of a map with a representation of the terrain and updated, high-resolution orthophotos.

### 2.3. Methodological Approach

**Figure 2:**
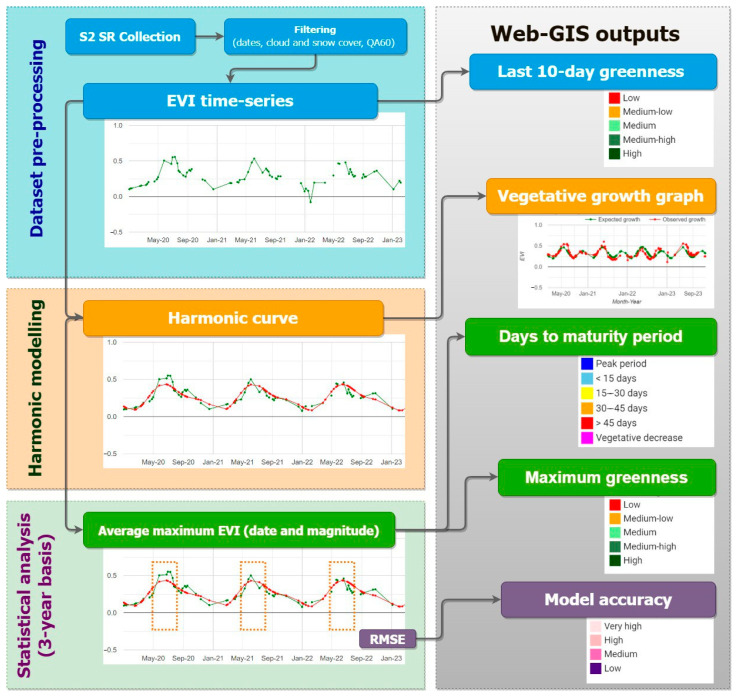
Methodological flowchart of the research. Orange dotted rectangles highlight the maturity period.

Considering the needs expressed by the panel, we defined a workflow for generating the user interface’s outputs in GEE (Figure 2). The three main methodological steps are (a) data pre-processing, (b) harmonic modeling, and (c) statistical analysis. The data pre-processing step aims at Sentinel-2 data selection and filtering and EVI time-series calculation. The EVI data are used for the subsequent harmonic modeling and to map the most recent vegetative status through an average calculation of the last 10 days. The second step aims to reconstruct the time series using the harmonic modeling approach. The output is used for the subsequent analysis and to build a vegetative growth graph for users to compare modeled and observed EVI-based growth curves. The third step aims to calculate the relevant EVI statistics considering the modeled and the observed curves. This statistical analysis based on three years generates three key output maps: the days remaining to the next maturity period, the average maximum greenness, and a model accuracy metric. In the following paragraphs, the aforementioned methodological steps are described in detail. 

#### 2.3.1. Satellite Data Pre-Processing

The complete archive of Sentinel-2 data is freely accessible through GEE. The spatial resolution (ranging from 10 to 60 m depending on the bands) and temporal resolution (approximately four days on average, depending on the location) make these data particularly efficient for monitoring vegetation [25]. Praterie utilizes Sentinel-2 data from the Harmonized version. In January 2022, there was an upgrade in Sentinel-2 products. Sentinel-2 scenes with the processing baseline 04.00 or above experienced a DN (digital number) range shift. The Harmonized collection aligns the data in newer scenes to match the range of older scenes.

Our application relies on historical data to estimate the phenology of the upcoming vegetation cycle. The three previous calendar years (i.e., 2020–2023, when this research was developed) provide a solid basis for identifying the seasonal vegetation patterns without considering year-to-year variations (influenced by specific weather conditions) or large-scale temporal variations (e.g., decade-scale variations influenced by climate change).

Initially, the data undergo filtering and correction processes to utilize only the highest-quality data. In the first step, Sentinel-2 data are filtered on the basis of time range and cloud coverage (i.e., cloudy pixel percentage), excluding images with a coverage percentage equal to or greater than 20%. Additionally, the Sentinel-2 Scene Classification Map (SCL) band is utilized to exclude saturated or defective pixels, as well as those representing cloud shadows, clouds, cirrus clouds, or snow (corresponding to classes 1, 3, and 7 to 11 in the SCL band, respectively). After obtaining the set of highest-quality Sentinel-2 data, the *EVI* is calculated with the following formula [40]:

$$EVI=G\cdot \frac{NIR\;-\;RED}{NIR\;+\;C_{1}\cdot RED\;-\;C_{2}\cdot BLUE\;+\;L}$$

“*NIR*”, “*RED*”, and “*BLUE*” represent the reflectance in the near-infrared, red, and blue bands, respectively, (i.e., bands 8, 4, and 2, respectively, of Sentinel-2). The coefficients adopted in the *EVI* formula are *L* = 1, *C*_1_ = 6, *C*_2_ = 7.5, and *G* = 2.5 [41,42]. Compared to NDVI, the *EVI* algorithm minimizes the effect of atmospheric conditions and adjusts for canopy background signals, thereby enhancing its sensitivity to fluctuations in regions with high biomass [43,44]. The *EVI* was also chosen because of its ease of interpretation and because it has already been used for grassland monitoring and management [26]. Furthermore, it consistently finds application in empirical connections with biomass measurements in pastures and meadows, establishing the foundation for estimating grass productivity [45,46,47,48,49,50,51].

#### 2.3.2. Harmonic Modeling

Harmonic analysis, also known as spectral or Fourier analysis, breaks down a periodic event that varies with time into a set of sinusoidal functions, each characterized by distinct amplitude and phase values [52]. These harmonic terms are combined to create a complex curve, and each component curve or term contributes to a portion of the total variance in the original time-series data set. This approach aims to produce a smoothed and continuous fitted time series of vegetation indices, effectively filtering out noise caused by outliers, missing data, or cloud cover effects [53,54]. Harmonic modeling has already been successfully employed to capture seasonal variations in vegetation, reduce noise, and fill in missing data, for instance, using NDVI [53] and in land cover classification studies (e.g., [55,56]). Harmonic modeling techniques have been devised in GEE, utilizing a linear combination of sine and cosine functions [57]. 

This study applied harmonic modeling to reconstruct and smooth the EVI time series of the three previous calendar years. The model application on the time series in GEE is very straightforward. It requires the definition of only one main parameter: the number of harmonics, which, in the case of vegetation modeling, is equal to the number of vegetation cycles per year. The grasslands of the study areas typically exhibit a pattern with two vegetative cycles and, consequently, two peaks: one in late spring/summer and the second at the beginning of autumn. For this reason, a model with two harmonics was employed. In GEE, an EVI harmonic model was developed for each 10 m pixel within the study area.

#### 2.3.3. Statistical Analysis

Once the harmonic models of the EVI were generated, they were used to calculate average vegetation indices values and their timing. Specifically, the maximum EVI peak was identified for each of the three years and the two annual peaks, and then the three-year average was calculated. Subsequently, the average date of the maximum EVI peak was determined for each annual peak, and the vegetative maturity period of grasslands was defined as the 12 days before and after that date. This interval was defined by analyzing several EVI time series graphs for representative grassland areas. 

The average maximum EVI values were classified according to five classes used for the layer visualization. These classes were defined iteratively by analyzing the frequency distribution in the study area and comparing the output classifications in representative grassland sites. The current date and the vegetative maturity period were used to dynamically calculate and map the days remaining or past the start of the maturity period. This value was classified into six classes to provide timing information about grassland vegetative development and support grazing activity management.

Root mean square error (*RMSE*) of the residuals was utilized to assess the accuracy of the three-year harmonic modeling of the EVI. The difference (residuals) between the observed EVI values and the estimated values obtained through harmonic models was calculated for each pixel within the area of interest. Subsequently, the standard deviation of these residuals, which represents the *RMSE*, was computed. The *RMSE* formula is as follows:

$$RMSE=\sqrt{\sum_{i=1}^{N}\frac{{\left({Predicted}_{i}-{Actual}_{i}\right)}^{2}}{N}}$$

Higher mean values indicate a more significant disparity between the model’s predictions and the observed NDVI data. Therefore, the *RMSE* indicates how closely the predicted EVI pattern from the harmonic model aligns with the observed EVI trend. *RMSE* values were classified according to four classes defined iteratively by analyzing the *RMSE* frequency distribution and visually comparing the modeled and observed EVI curves in representative grassland sites. 

### 2.4. Application Development

The Praterie app was encoded in JavaScript and developed using GEE. The app uses the current date to retrieve Sentinel-2 images available after atmospheric filtering for the three previous years. After calculating the EVI based on the acquired dataset and applying harmonic modeling, three main layers of estimated vegetation growth are calculated: (1) the estimated number of days remaining until the next maturity period (i.e., days to the next maturity period), (2) the average maximum expected greenness for the next peak (i.e., maximum greenness), and (3) the accuracy of the harmonic model based on the RMSE (i.e., model accuracy). From 30 November to 31 July, the estimated parameters for the first cycle of the current year are shown. The estimates for the second cycle are shown for 31 July to 30 November. The three layers are exported into a GEE asset to increase the visualization speed by the app users. Thus, in the present code version, this step requires a manual asset update at the beginning of each year. Weather conditions heavily influence actual vegetation growth. As a result, the actual growth curve may deviate considerably from the estimated growth based on historical data. For this reason, the average EVI of the previous 10 days is also calculated and shown in a layer (i.e., last 10-day average greenness), contingent upon the availability of images that fulfill the specified filtering criteria. 

Specific functions are implemented in the GEE code to enable users to generate the EVI graph’s time series by clicking on a point on the map or drawing a rectangle or a polygon. A “find my location” function was integrated to allow users to center the map on the current position. For this functionality, it is necessary to authorize the app to use the device’s location.

Praterie was developed in two versions. One is specifically designed for desktop usage, and the other is for smartphones, with reduced functionality compared to the desktop version to improve the app’s functioning on mobile devices.

## 3. Results

### 3.1. The Main Interface

**Figure 3:**
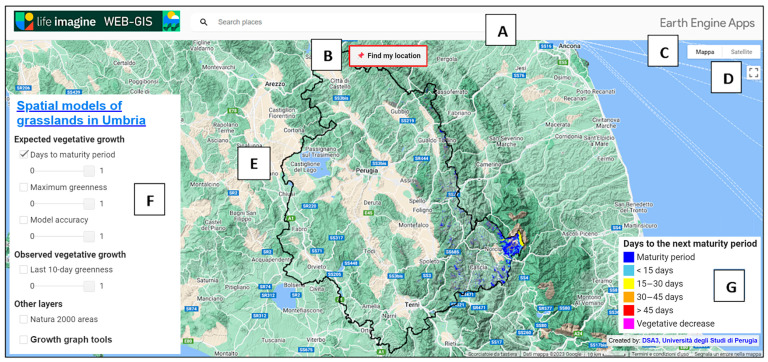
The default main interface of the Praterie app. It includes a search bar to find the location of interest (**A**), a tool to find the user’s location (**B**), buttons to change the background map (**C**), a button to change the display to full screen (**D**), the borders of the Umbria region (**E**), a panel including all information levels (**F**), and the corresponding legends on the right (**G**).

The Praterie app is accessible from https://ee-mvizzari.projects.earthengine.app/view/imaginegrasslands (accessed on 24 January 2024). Access to the app, as is usual for GEE apps, does not require any additional software installation and is completely free. Moreover, it offers the advantage of not requiring a GEE account, ensuring all users’ access directly through a web browser. Figure 3 shows different panels containing levels and legends, the location search bar, and a geolocation tool. As the application runs, the main window is centered on Umbria and regional borders become visible. Additionally, the main window displays the “days to the next maturity period” layer, updated to the current date.

The automatically displayed background map at the start of the Praterie app is a Google Maps topographic base map, including the relief. The background map can be modified using dedicated buttons: “map” and “satellite.” The “map” button allows the default two-dimensional map of Google Maps to be set as the background, and the relief effect can be turned on or off by toggling the corresponding flag. The “satellite” button, on the other hand, allows satellite imagery to be displayed as the background, providing a photorealistic representation of the surfaces. The satellite view can be beneficial for inferring information about the geographic features of the territory, land cover, and vegetation. The acquisition date of the available images is indicated in the bottom right corner of the main window. 

The “find my location” button allows user to locate their position on the map with a GPS-equipped device. This function is especially beneficial when the user is in his area of interest, significantly reducing the time and effort required for manual map search.

In the left panel, the user can select the layers they want to display on the main screen by clicking the checkbox. Once the checkbox is activated and the visualization of a layer is enabled, the corresponding legend automatically appears in the bottom-right corner. Furthermore, the user can adjust the opacity of one or more layers to view them overlaid. 

Users can access the tool in the same panel to build a vegetation growth chart for a location of interest. The chart is interactive and is displayed in a new panel that appears in the bottom right corner after the loading time. Once generated, the chart displays the observed vegetation growth (red line) and the estimated vegetation growth (green line) obtained through the application of the harmonic model. The *y*-axis represents EVI values ranging from −1 to +1, whereas the *x*-axis represents dates in the month–year format (MM–YY). The data in the graph range from three years before the most recent date with available images. In the case of a rectangle or polygon used for defining the location of interest, the graph shows the average values for the area covered by the drawn geometry. As found in other works (e.g., [25]), it should be noted that the algorithms employed for cloud masking in Sentinel-2 images do not always yield precise outcomes. As a result, atypical points, usually related to residual clouds [25], may appear in the time series graphs.

In the mobile version, adjusting the transparency of layers is not allowed, and the tool for generating a vegetation growth graph is not supported. Furthermore, all panels are scaled down to fit the smaller screen and the legends are smaller, potentially providing less detailed information than the desktop version.

### 3.2. Layer Visualization 

**Figure 4:**
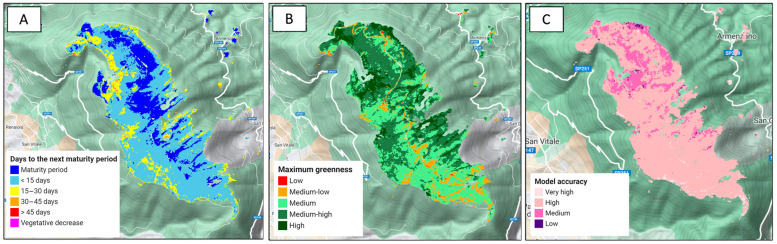
Example of the three layers ((**A**) days to the next maturity period; (**B**): maximum greenness; (**C**) model accuracy) generated for the first vegetation cycle (late spring–summer) of 2023 for the Monte Subasio area, one of the Special Areas of Conservation included in the Natura 2000 network.

The expected vegetative growth layer group includes three layers (Figure 4). The “days to the next maturity period” layer represents the number of days remaining until the start of the next vegetative maturity period. The values are divided into six categories, with four categories representing 15 day intervals from 0 to greater than 45 days, one category identifying areas currently within the temporal window of the maximum greenness period, and the last category identifying areas where vegetation is declining. The “maximum greenness” layer includes values divided into five categories. Considering that the areas of interest are all vegetated and classified as grasslands, maximum EVI values of less than 0.3 are categorized as “low greenness”, “medium–low” for values of 0.3 to 0.4, “medium” for values of 0.4 to 0.5, “medium–high” for values of 0.5 to 0.6, and finally, “high” for EVI values of more than 0.6. The “model accuracy” layer is represented by the RMSE values for the harmonic modeling over the previous three calendar years. The RMSE values are divided into four accuracy categories: “very high” for values of less than 0.05, “high” for values of below 0.1, “medium” for values of 0.1 to 0.15, and “low” for values of more than 0.15. The “observed vegetation growth layer” group includes the “last 10 days greenness” layer, which is represented by the mean EVI values of the previous 10 days. This layer is available or not based on the cloud cover of the previous 10 days. The representation of this layer follows the categorization of values used for the “maximum greenness” layer. 

**Figure 5:**
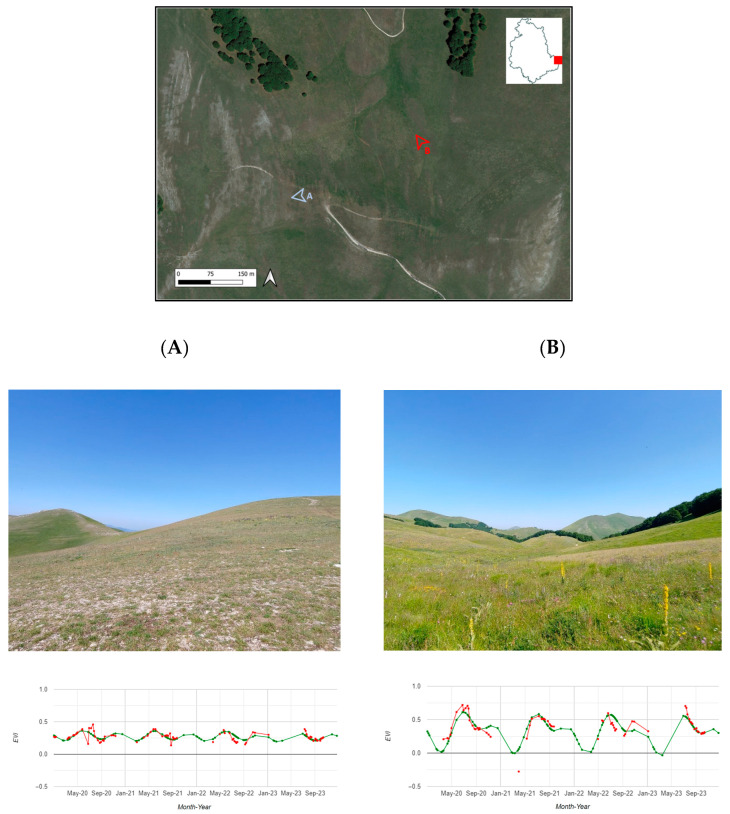
Field observations on 10 July 2023 in areas (**A**,**B**) located near Monte delle Rose (Norcia, PG, Italy) at an altitude of approximately 1700 m a.s.l. The vegetation growth graphs, displaying observed EVI values in red and expected EVI values in green, of both areas extracted from Praterie are also shown.

Here, we report two exemplificative field observations showing the functionalities of Praterie. The first area (Figure 5A) comprised sparse herbaceous vegetation with exposed rock, consistent with the xerophilous facies in Annex I habitat 6210. It was characterized by medium–low and low maximum greenness values, indicated by average maximum EVI values of less than 0.4, as shown by the vegetation growth graph generated for the observation area (Figure 5A). On the other hand, the second observation area (Figure 5B) is characterized by thick swards dominated by *Nardus stricta*, classifying it as Annex I habitat 6230*. This area exhibited high and very high average maximum EVI values of more than 0.5, as highlighted by the vegetation growth graph (Figure 5B). The photos and data from the app were obtained for both areas during the expected maturity period.

### 3.3. Descriptive Statistics and Model Accuracy

Table 1 reports the descriptive statistics for key variables (the average maximum EVI values—“EVI max” and their timing—“EVI max DOY”) and the accuracy results (RMSE) calculated on a three-year basis (2020–2022) for the first (FC) and the second (SC) cycles from the harmonic models. The “EVI max” mean for each annual peak, with values of 0.47 and 0.39 for FC and SC, respectively, and the low standard deviations (0.1 for both cycles), suggest that, as expected due to the local climate, the average greenness in the first cycle tended to be remarkably higher compared to the second one. The “EVI max DOY” statistics suggest that the greenness for the FC occurred on average towards the end of May (mean DOY = 149.6), with a standard deviation of approximately 20 days, providing a significant temporal clarification on the season of maximum vegetative activity in the grasslands. On the other hand, the maturity period for the second cycle occurred on average towards mid to late October (mean DOY = 249), with a standard deviation of approximately 24 days. Of course, the spatial variability of these values was mainly influenced by the elevation and the aspect. 

The RMSE results are quite promising and demonstrate the models’ overall performance, showcasing a generally good level of accuracy with an average RMSE of 0.07, a standard deviation of 0.06, and a maximum value of 0.33. The areas corresponding to the various models’ accuracy classes are reported in Table 2. The “very high” accuracy level was seen in an area of about 99 square kilometers. This indicates that the harmonic models were extremely reliable in these regions, accounting for about 26% of the total area. The “high” accuracy level was observed in a significantly larger area of about 238 square kilometers, which made up about 62% of the total area. This suggests that, although there may have been some small deviations from the actual values, the models’ performance was still largely accurate and dependable in these areas. The combined “very high” and “high” accuracy levels covered about the 88% of the total area, demonstrating the overall effectiveness of the harmonic models. The “medium” accuracy level, seen in an area of about 39 square kilometers, indicated a moderate level of accuracy. Although the models’ predictions or measurements in these areas were reasonably reliable, they may not always have been entirely accurate, accounting for about 10% of the total area. The “low” accuracy level was found in a small area of about 7 square kilometers, representing only about 2% of the total area. This suggests that the models’ performance in these areas was less reliable and that their predictions may often have deviated considerably from the observed values.

## 4. Discussion

Grazing management for open habitats necessitates understanding how grazing impacts species composition and diversity and a deep and thorough comprehension of the mechanisms that shape their ecological functioning [13,58]. Crucial for sustainable grazing management is the accurate and timely allocation of grazing areas based on animals’ nutritional needs and vegetation availability [16]. Indeed, grazing by animals is recommended when pastures exhibit a specific structure, primarily defined by the height and density of forage, ensuring the grazing of only grass leaves [58]. It is imperative to implement special programs to promote more sustainable grazing management to counteract the degradation trends and preserve biodiversity in open landscapes [1,13]. Through the adoption of precise and sustainable grazing practices, the optimization of grassland utilization becomes achievable [16,58]. These efforts play a crucial role in safeguarding the environment, maintaining the delicate balance of ecosystems, and meeting the European Community’s conservation targets in the Habitats Directive framework. However, there is often a lack of high spatiotemporal resolution data regarding various crucial aspects of grazing, including herbage mass and growth rate, as well as herbage quality and the botanical composition of the grazing area [16]. Comprehensive information about these aspects is paramount in attaining precise, efficient, sustainable grazing practices. In recent years, significant technological advancements have revolutionized data acquisition on herbage availability and the monitoring of animal movement for more efficient and sustainable grazing animal stocking [16,59]. Despite this, the widespread integration of these practices into current farming methods has been limited [22]. For the sustainable, timely, and precise utilization of grassland, it is essential to determine the ideal time for the grassland to be grazed, the appropriate moment to move the grazers to another area, and the optimal grazing load to ensure both effective plant recovery and preservation of the habitat’s functional conditions [16]. In this specific context, there is a clear and pressing necessity to foster the development of novel and pioneering precision decision-making tools tailored to the needs of farmers [22,60]. 

Our application addresses this demand by offering various advantages. It is free and accessible to everyone, with no requirements of technical skills or prior knowledge in using applications or geospatial data. Its intuitive interface ensures effortless navigation, allowing users to easily visualize the condition of grasslands both during the upcoming vegetative maturity period and within the last 10 days leading up to the current date. Therefore, our application offers a high level of usability. However, although it provides easy access and visualization of grassland status, it does not directly incorporate livestock-related data, a relevant feature in other decision-support systems for grassland management (e.g., [23,29]). However, it has the potential for integration with other real-time herd-monitoring tools. Sophisticated grazing management involves precise control of animals’ location, grazing habits, and movement [16]. For example, farmers can use GPS receivers and sensors to locate and monitor individual animals, track their movement patterns, and understand group dynamics and pasture utilization [16]. One of the current innovative digital tools is virtual fencing, which eliminates the need for physical fences and can facilitate biodiversity-friendly grazing management [16,61,62]. This technology allows the farmers to precisely control animals’ location, directing them to specific areas at optimal times and keeping them away from more vulnerable habitat areas [16]. In addition, although other grassland decision support systems focus on individual plots for each agricultural business (e.g., [24,25]), our tool can offer a comprehensive regional-level overview of grassland conditions.

The elevation threshold employed to delineate the study region facilitated the incorporation of an area primarily comprising pastureland. Nonetheless, certain sections within this region contain meadows, resulting in notably poor accuracy in harmonic modeling due to the irregularities caused by mowing events creating an uneven curve pattern. In these easily recognizable areas, the “days to maturity” and “maximum greenness” layers could provide inaccurate information, highlighted by the low accuracy of the model output. However, the observed EVI curve can provide users with interesting information about the dates of mowing events and the vegetation growth rates. The observed data about the last 10 day average greenness can also be beneficial, informing users on the current vegetative state of the meadows.

As performed in previous studies [53,63,64], applying harmonic modeling to vegetation index time-series data demonstrated significant utility in capturing seasonal patterns and cyclical changes in vegetation dynamics. This step is essential because, when utilizing data from optical sensors, the acquisition of images and information through remote sensing is often significantly affected by cloud cover and weather conditions. This approach allowed us to model EVI time-series data and identify and analyze the periodic patterns in vegetation growth, maturity, and decline. The use of harmonic modeling provides several advantages over other methods. Firstly, it effectively reduces the dimensionality of the time-series data, making them more manageable and less computationally intensive. Secondly, it allows for identifying annual patterns, which can be crucial in understanding the dynamics of different grassland ecosystems. Thirdly, harmonic modeling is less sensitive to noise and missing data, which are common issues in remote sensing datasets. However, when time-series data become scarce or significant inter-annual fluctuations are observed, the harmonic model accuracy decreases significantly.

Our application provides information regarding grasslands with high spatial and temporal resolution using EVI time-series data. However, the results displayed in our application were derived from historical data, and therefore, our projections reflect past (although recent) vegetation trends. Vegetation indices such as the EVI and the NDVI have been related to grassland productivity and used to model the primary production of these surfaces. However, these relationships vary based on local factors such as soil, topography, vegetation composition, and climate [45,46,47,48,49,50,51,60]. Thus, it would be interesting to integrate vegetation productivity models based on remote-sensed data and calibrated with on-field surveys that could allow for real-time adjustment of the detected vegetation trends based on the current season’s weather conditions, particularly temperature and precipitation. In this context, ERA5-Land constitutes an appealing climate reanalysis dataset. It offers various benefits, like land and atmospheric variables at a satisfactory spatial resolution (about 9 km) and a temporal resolution of hourly updates [65]. However, there is occasionally a delay in updating GEE catalogs. Until a few months ago, in GEE, these data used to be updated with a delay of approximately three months from the current date. Fortunately, there have been recent improvements, and the latency has been reduced to about 10 days. However, the official Copernicus Climate Data Store offers a latency of approximately five days for the same data. Moreover, the ERA5 data should be upscaled using the proper approaches to adapt them to the local variability conditions. In addition, GEE’s data catalog might not continuously be updated with adequate timing, which could affect the availability of the most recent data for specific applications.

Remote sensing enables the monitoring of grassland phenology on a large scale, offering a comprehensive view of vegetation growth stages and patterns [66]. Various satellite-based vegetation indices, such as the NDVI and the EVI, detect phenological changes over time. Modeling these changes helps predict future grassland dynamics under different environmental scenarios, thereby contributing to sustainable grassland management strategies [67]. At this stage, the Praterie app uses a simplified approach to identify and forecast the grassland maturity period. Future developments can include advanced modeling approaches to derive more affordable and accurate phenological metrics [68,69].

Overall, the GEE platform provides powerful functionalities for easy web app development. It empowers developers to create interactive apps based on geospatial data using Python and JavaScript. Moreover, GEE offers an integrated, user-friendly code editor, making the development process even more convenient. The GEE code developed in this research can be a basis for future developments to improve the quantity and quality of information available for grassland conservation and management. However, it is essential to note that the GEE app access, as with most Web-GIS tools, requires an internet connection, as it relies on online resources and data. All the functionalities of Praterie are enabled through JavaScript code, and their proper functioning depends on an active internet connection during usage. This aspect may limit its practical use in the field, particularly in remote regions such as high-altitude secondary grasslands. Moreover, the need for a manual update of the three main layers at the beginning of each year due to the impossibility of automating it in GEE, even if it is a straightforward operation, is a drawback since it requires an intervention once a year. 

## 5. Conclusions

The GEE platform developed in this research allows for the easy visualization and investigation of different layers depicting the trends in grassland vegetation. It allows for areas with higher density and greenness to be identified in the next vegetation cycle and the estimated days to reach vegetation maturity to be determined at a spatial resolution of 10 m. The interface is versatile and could serve diverse applications. Notably, it can be a support for institutions and public bodies in the development of site-specific grazing plans. Moreover, local herders and farms could utilize its capabilities, integrating it with other cutting-edge technological tools like virtual fencing. 

Currently, Praterie is geographically limited, as it is designed explicitly for the grassland areas of the Umbria region in Central Italy. Nevertheless, we believe its potential for expansion is substantial, as it can be readily extended to cover broader geographical regions. At this stage, the Praterie app uses a simplified approach to identify and forecast the grassland maturity period. In this regard, future developments can include advanced modeling approaches on time-series data to derive more accurate phenological metrics and information about grassland productivity. 

Although there is room for improvement, our app represents a significant step towards fostering innovation and progress in this field. Indeed, our application can contribute to the innovation and digitalization process within sustainable livestock farming while also functioning as a decision support system for managing habitats of biodiversity conservation importance.

## Figures and Tables

**Table 1:** Descriptive statistics, including mean, minimum, maximum, and standard deviation, computed for the three-year average maximum EVI values (2020–2022) of each pixel and their corresponding timing for the first vegetative cycle (FC) and the second vegetative cycle (SC).

	Mean	Min.	Max.	Std. Dev.
EVI max—FC	0.47	0.03	1	0.1
EVI max—SC	0.39	0	1	0.1
EVI max DOY—FC	149.57	93.67	209.3	20.41
EVI max DOY—SC	291.09	245.3	326.3	23.7
RMSE	0.07	0.01	0.33	0.06

**Table 2:** Area and related percentages of the models’ accuracy classes (2020–2022).

Accuracy	Area (kmq)	Area (%)
Very high	98.81	25.8
High	238.40	62.2
Medium	38.85	10.1
Low	7.42	1.9

## Data Availability

All remote sensing data used in the research are openly available in the Copernicus Open Access Hub (https://scihub.copernicus.eu/, accessed on 26 January 2024) and within Google Earth Engine.
